# Model averaging strategies for structure learning in Bayesian networks with limited data

**DOI:** 10.1186/1471-2105-13-S13-S10

**Published:** 2012-08-24

**Authors:** Bradley M Broom, Kim-Anh Do, Devika Subramanian

**Affiliations:** 1 Department of Bioinformatics and Computational Biology, UT MD Anderson Cancer Center, Houston, Texas 77030, USA; 2 Department of Biostatistics, UT MD Anderson Cancer Center, Houston, Texas 77030, USA; 3 Department of Computer Science, Rice University, Houston, Texas 77005, USA

## Abstract

**Background:**

Considerable progress has been made on algorithms for learning the structure of Bayesian networks from data. Model averaging by using bootstrap replicates with feature selection by thresholding is a widely used solution for learning features with high confidence. Yet, in the context of limited data many questions remain unanswered. What scoring functions are most effective for model averaging? Does the bias arising from the discreteness of the bootstrap significantly affect learning performance? Is it better to pick the single best network or to average multiple networks learnt from each bootstrap resample? How should thresholds for learning statistically significant features be selected?

**Results:**

The best scoring functions are Dirichlet Prior Scoring Metric with small *λ* and the Bayesian Dirichlet metric. Correcting the bias arising from the discreteness of the bootstrap worsens learning performance. It is better to pick the single best network learnt from each bootstrap resample. We describe a permutation based method for determining significance thresholds for feature selection in bagged models. We show that in contexts with limited data, Bayesian bagging using the Dirichlet Prior Scoring Metric (DPSM) is the most effective learning strategy, and that modifying the scoring function to penalize complex networks hampers model averaging. We establish these results using a systematic study of two well-known benchmarks, specifically ALARM and INSURANCE. We also apply our network construction method to gene expression data from the Cancer Genome Atlas Glioblastoma multiforme dataset and show that survival is related to clinical covariates age and gender and clusters for interferon induced genes and growth inhibition genes.

**Conclusions:**

For small data sets, our approach performs significantly better than previously published methods.

## Introduction

In the last ten years there has been a great deal of research published on learning Bayesian networks from data [1-4]. Most of the work in structure discovery in Bayesian networks has focused on designing computationally tractable procedures for searching the space of networks, and on devising metrics for scoring networks during search. However, the problem of determining the quality and robustness of learned structures in the context of limited data remains largely open. Structure learning algorithms are known to be unstable — small changes in training data can cause large changes in the learned structures. As Bayesian network learning begins to find serious application in biology [5], there is an increasing need for learning protocols that can not only discover network features, such as edges and Markov blankets, from small amounts of data, but can also determine an accurate confidence estimate for those features. In many biological and clinical settings, the size of the dataset may be severely limited, either by high cost or by the limited number of cases from which data can be collected (for instance, people affected by a rare disease) [6]. In such settings, it is worthwhile to use substantial amounts of computation in order to achieve as accurate a result as possible. Model averaging using bootstrap replicates (that is, bagging) with feature selection by thresholding [7] is a widely used solution [8]. Unfortunately, there is no established method for determining feature-selection thresholds. There is no prescription for the choice of bootstrap technique: the ordinary non-parametric bootstrap [9], a bias-corrected version of the non-parametric bootstrap [10], or the more continuous Bayesian bootstrap [11]. Whether the choice of bootstrap method interacts with the choice of scoring function is unknown, as is the number of bootstrap replicates needed to achieve a certain level of confidence in the final model.

In this paper, our goal is to understand the interactions between choice of bootstrap method and scoring function, size of training data and number of bootstrap replicates as well as the choice of feature-selection threshold on averaged models. Unlike linear regression, for which closed form expressions for the performance of model-averaging procedures can be derived [12], our model class is too complex to permit the analytical derivation of quality guarantees. We therefore use experiments on two moderately sized Bayesian networks with known structure (ALARM and INSURANCE) to systematically study the effects of varying the bootstrap technique, the amount of training data, the number of bootstrap replicates, as well as the scoring function for network learning. We design a variant of permutation testing to automatically select thresholds for feature selection in bootstrap averaged (bagged) models and construct a loose upper bound for the number of bootstrap replicates needed for stable estimates of feature probabilities.

**Figure 1:**
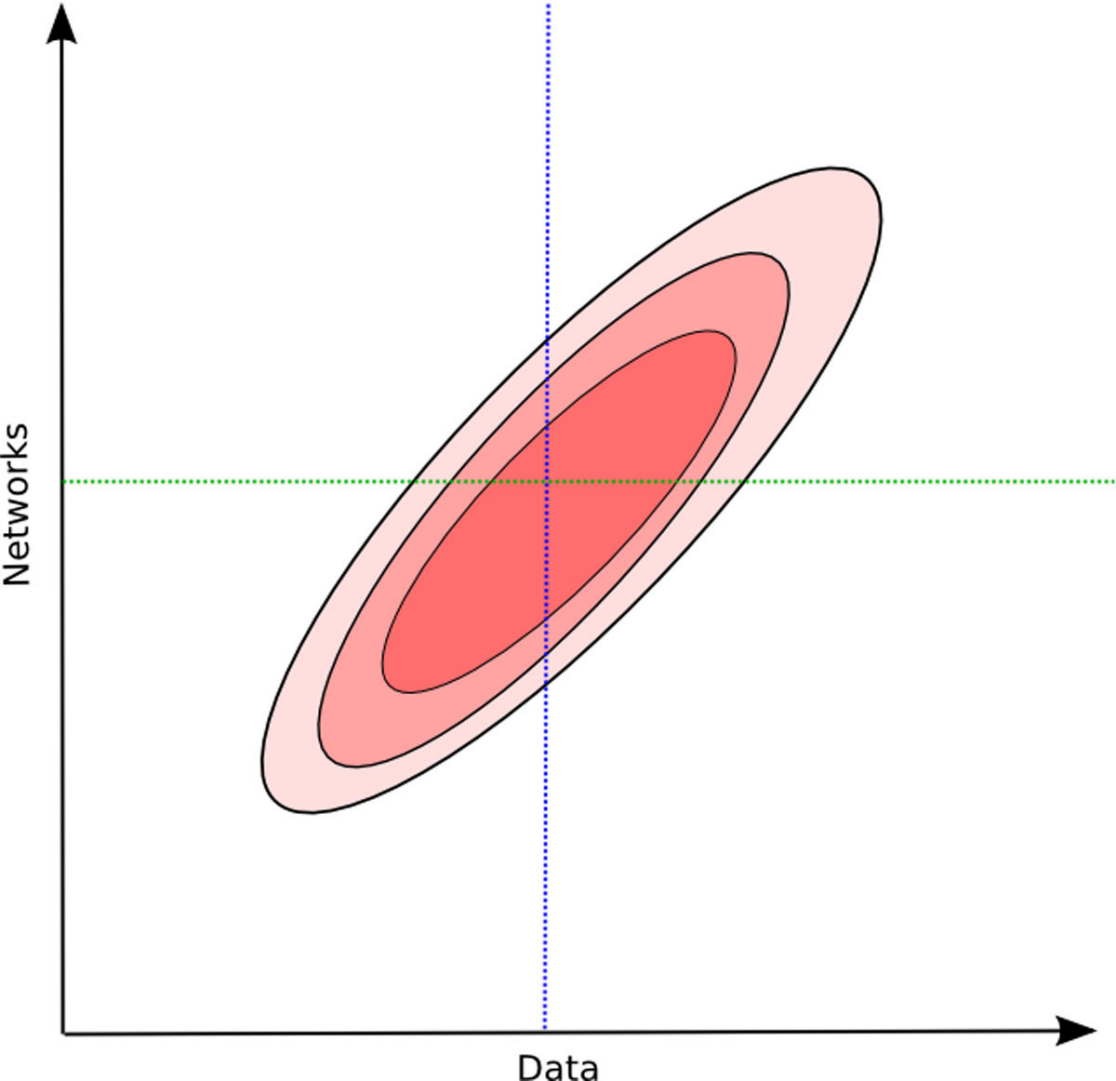
**Bayesian network search space** The search for Bayesian networks guided by data. Contours represent likelihood of structures given data.

The variance in the structure of learned networks comes in part from the data, which is usually a single realization from some unknown true model. This is schematically depicted in Figure 1, where possible data sets sampled from the true model lie on the *x* axis, and potential Bayesian networks on the given set of variables are on the *y* axis. The contours denote regions in which the posterior probability of the network given the data are the same. Given a single data set, exploration in the structure space is completely determined by the parameters of the search algorithm. This is denoted by the vertical dotted blue line in Figure 1.

When the amount of data is small relative to the size of the model, the posterior probability of the model given the data is not sharply peaked around a single model [13]. To correctly assess the probability of a structural feature (for instance, an edge) in the model, we need to average over the entire ensemble of networks with similar posterior probabilities. If it is reasonable to assume that all high-scoring structures are equally good, we have the probability of a feature *e* given data  to be(1)

or we can compute Bayesian posterior probabilities(2)

where we weight each feature in proportion to the posterior probability of the structure  in which it occurs.

For large networks, the direct estimation of  is intractable. Structure learning algorithms approximate the computation in Equations 1 or 2 by only considering high scoring structures encountered during search. These structures tend to be quite similar to one another, as evidenced by the fact that feature (such as, edge) probabilities computed from them are usually close to 0 or 1. Computing feature probabilities by MCMC sampling over structures drawn from the posterior distribution  has been proposed by Madigan and York [14] and refined by Giudici et al. [15]. These techniques do not scale to large networks, and have been shown to be slow-mixing [13].

A different approach to estimating feature probabilities is to sample in different sections of the structure space by perturbing the data set . In effect, we follow the horizontal green dotted line parallel to the *x* axis in Figure 1. By taking bootstrap resamples from the original dataset, learning a single high-scoring Bayesian network from each resample, and averaging feature probabilities over these networks, we can obtain feature probability estimates from a more diverse population of structures. This is the motivation behind Algorithm 1 shown below, proposed in [4]. A final network is assembled by including only those structural features that have estimated probabilities above a pre-specified threshold *t*, which is given as an input to the algorithm.

**Figure 2:**
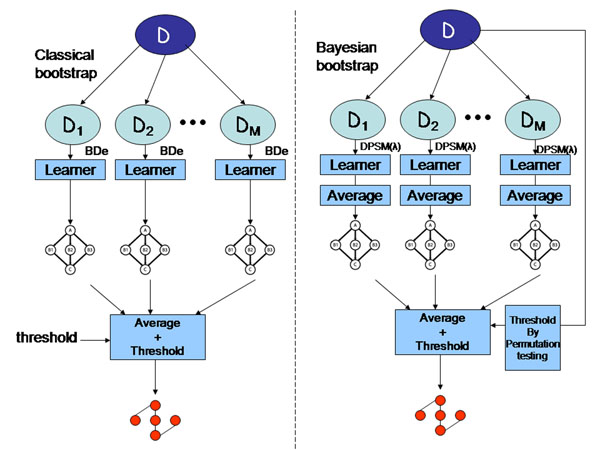
**Bayesian model averaging strategies** On the left is the model averaging protocol of [4], on the right is our proposed model averaging strategy. The three key differences are the choice of scoring function, learning multiple models from each bootstrap resample, and automatic determination of the probability threshold.

The practical application of this algorithm, particularly in the context of limited data requires answers to many questions. Specifically:

• How many bootstrap resamples *M* are required to obtain stable estimates of the feature probabilities in bagged models?

• Which scoring functions *F_s_* work best with bagging? Friedman et al. [4] only used BDe as a scoring function in their bootstrap experiments. Hartemink et al. [16] offer experimental evidence that that BDe outperforms MDL/BIC in a bagging context. It is an open question how scoring functions such as DPSM compare to BDe.

• Is the bias in bagged Bayesian networks arising from the discreteness of the bootstrap method [10] a significant problem? If so, would a continuous version of bootstrap, like the Bayesian bootstrap [11] be a better choice for bootstrap resampling than the ordinary discrete bootstrap?

• How do we select thresholds for learning statistically significant features over averaged models? Does the choice of threshold depend on the scoring function, problem domain, sample size, and bootstrap resampling technique?

• Should a single high scoring structure be learned from a bootstrap replicate (as shown in Algorithm 1), or an averaged ensemble of high scoring structures (double averaging, as shown in Figure 2)?

The paper is organized as follows. In the next section, we review the problem of learning the structure of Bayesian networks from data, and describe the state of the art in scoring functions, search algorithms, and bootstrapping techniques. In the following section, we present an extensive experimental study that explores the space of model-averaging strategies in the context of the ALARM network. We then describe our technique for automatically selecting a probability threshold for feature selection. Our threshold selection criterion is designed to minimize the number of false positive features in bagged models. We conclude with a characterization of an effective model-averaging strategy in contexts with limited data.

## Learning the structure of Bayesian networks

### Bayesian networks

Bayesian networks are a compact, graphical representation of multivariate joint probability distributions on a set of variables. The graph structure reflects conditional independence relationships between the random variables. Formally, a Bayesian network for a set **X** = {*X*_1_,…, *X_n_*} of *n* discrete random variables is a pair  where  is a directed acyclic graph whose vertices represent the random variables *X*_1_,… , *X_n_*, and whose edges represent direct dependencies between these variables. Θ represents the set of conditional probability distributions of the form Θ*_X_i__*_|__**U***_i_*_ = *P*(*X_i_*|**U***_i_*), 1 ≤ *i* ≤ *n*, where **U***_i_* denotes the parents of variable *X*_i_ in the graph *.* The joint probability distribution **P**(*X*_1_,…, *X_n_*) encoded by  can be reconstructed as the product of the individual conditional probability distributions in Θ:

### Learning Bayesian networks from data: scoring functions

The problem of learning a network from data is normally posed as an optimization problem: Given a set  of instances drawn from a multivariate joint probability distribution **P**(**X**), find a network  which maximizes the posterior probability of the network given the data:

The prior over network structures has two components: a discrete probability distribution  over graph structures , and for each possible graph, a density measure  over possible values of the parameter Θ. The simplest, and most common choice for both the prior  over graphs as well as the parameter distribution , is the uniform distribution. Assuming that the data is sampled *i.i.d.*, the likelihood of the data given a network  is

The posterior probability  is called a scoring metric or scoring function for Bayesian network learning algorithms. By marginalizing over all choices for the parameter distribution Θ, we can write  as

The scoring function  simplifies under the assumption of uniform structure and parameter priors to the following, called the K2 metric in [17], and the UPSM score in [18]:

where *r_i_* is the number of values that *X_i_* takes, and *q_i_* is the number of values that the parents of *X_i_* in  take. *N_ijk_* is the number of instances in  in which variable *X_i_* has its *k^th^* value, and its parents take their *j^th^* value, and .

A generalization of the above scoring metric uses Dirichlet priors (*λ* >= 1) for . The parameter priors for each variable are considered independent of one another, an assumption called global parameter independence in [13],

In addition, the parameter modularity assumption is made [19]. For two graph structures  and  such that the parents of node *X_i_* are the same in both graphs, we have

The graph structure parameter prior  is assumed to satisfy structural modularity; that is, it can be factored as a product of distributions over the possible parent sets of node *X_i_* in the graph. Under these assumptions, the posterior probability of the graph structure  can be written in fully factored form as

The scoring function with these assumptions reduces to

where  is the Dirichlet distribution order for variable *i* with value *k* and parent value *j*, and . When the Dirichlet orders for all sets of parameters  are set to a constant *λ*, the score is called DPSM [18]. When we assume , it is called the Bayesian Dirichlet (BDe) metric [17]. The choice of  is critical, particularly for small data sets. If these parameters are large, making  dominate the *N_ijk_* values, the available data has less influence in determining the space of structures explored.

There is another family of scoring functions based on maximizing the likelihood function . The first among these is the BIC score, also known as the Schwartz criterion, which is derived from the Laplace approximation to the logarithm of  ( [20], page 64) and defined by

where  is the maximum likelihood estimate of Θ given  and *D*, *d* is the number of free parameters in Θ, and *N* is the size of the training sample. The number of free parameters in Θ is

where *r_i_* is the number of values that variable *X_i_* can take, and *q_i_* is the number of values that the parents of *X_i_* in  can take. Direct maximization of  yields overfitted models, because the likelihood of the data given  and Θ can be increased by increasing the complexity of  (that is, by adding an edge). The second term  can be seen as a penalty term to compensate for model complexity. Finding a structure  with the highest BIC score is equivalent to finding a structure with the highest approximate posterior probability given the data *D.* As the sample size *N →* ∞, the probability that the BIC criterion selects the true model approaches 1. However, with finite sample sizes, BIC tends to select models that are much simpler than the true model, due to the penalty term.

The MDL, or the Minimum Length Description scoring function has its origins in data compression and information theory. The best model for a data set , according to the MDL, is one that minimizes the sum of the length (in bits) of the encoding of the model itself, and the length of the encoding of the data given the model. When applied to Bayesian network structure learning, the MDL score is the sum of the description length of the graph , the description length of the conditional probability parameters Θ given , and that of the data  given  and Θ. The description length of the graph  is

where |*π_i_*| is the number of parents of *X_i_* in , and *n* is the number of variables *X_i_* in **X**. The description length of Θ is the product of the total number of independent parameters for specifying each conditional probability table, and the number of bits needed to encode each of these parameters for a data set of size *N.* Define

Finally, the number of bits needed to encode the data given the model is

Thus

Minimizing the MDL scoring function yields a model with the shortest description length. If we ignore the description length of the graph, , the MDL score equals the BIC score with reversed sign. The MDL scoring function favors graphs in which nodes have fewer parents, because it reduces the encoding length of Θ and .

To summarize, scoring functions belong to one of two families. Bayesian scoring functions, of which K2, DPSM, and BDe are examples, represent the posterior probability  of a structure  given data . The second family consists of complexity-penalized likelihood measures, of which BIC and MDL are examples. MDL can also be justified from an information-theoretic perspective. These scoring functions embody different assumptions about priors on the space of structures as well as priors on parameter distributions given the structures. There are few systematic studies in the literature to guide choice of scoring functions for specific applications. Below we show a comparative analysis of the performance of these scoring functions in the context of limited data.

### Learning Bayesian networks from data: local search algorithms

The problem of learning a network which maximizes  is usually posed as a combinatorial optimization problem. It is known to be NP-complete [21], so local search algorithms guided by scoring functions are used to find approximate solutions.

The local search algorithm used in our experiments is greedy hill-climbing with randomized restarts and Friedman’s sparse candidate algorithm [4] with *k* = 6 (maximum number of parents per node). Every network explored by the algorithm during the search is recorded, and no network is ever considered twice. Initially, the *k* most likely parents for each node, the candidates, are selected by scoring all networks with a single edge between two nodes. Denote by *K* the set of all networks in which the parents of every node belong to the set of *k* candidate parents for that node. A list of starting networks containing all networks in *K* with up to two edges is then generated. A starting network is picked at random from this initial list. From this starting network, all neighboring networks in *K* that have not been considered before and which differ by an additional edge, one less edge, or a reversed edge are evaluated. The highest scoring neighboring network, if its score is higher, replaces the current network. The search is continued until no new networks are generated or all generated networks score less than the current network. New sets of *k* candidate parents are then generated following Friedman’s algorithm, and the search is continued. New candidate parents sets are picked until a previously seen set of candidate parents is revisited, or *C* = 10 different candidate parent sets have been considered. Such searches starting from a randomly picked member of the initial network list are performed a total of *M*_1_ = 25 times. Another *M*_2_ = 25 such searches are performed starting from a network chosen randomly from all of those seen during the first *M*_1_ searches. Network features are determined from the highest scoring network(s) visited over all searches.

### Bootstrap and Bayesian bootstrap

The bootstrap is a general tool for assessing statistical accuracy of models learned from data. Suppose we have a data set , where each **x**^(^*^j^*^)^ is a vector of size *n* drawn from the cross product of the domains of variables *X*_1_,…, *X_n_*. The basic idea is to randomly draw datasets with replacement from , with each sample the same size as the original set, that is, *N.* This is done *B* times, producing *B* bootstrap replicates, as shown on the left in Figure 2. We learn Bayesian networks from each bootstrap resample. We can then analyze the networks generated over the *B* resamples; for example, producing means and variances on frequencies of structural features.

Each example in  is represented between 0 and *B* times among the bootstrap resamples. Thus one can can think of the standard bootstrap procedure as assigning each example in  an integer weight drawn from a multinomial distribution, representing its number of occurrences in the *B* resamples. The probability of not including a specific example in a resample is about 1/*e* ≈ 37%. Since an example contributes to the count *N_ijk_* in the scoring function; dropping examples biases the counts, and the structures that are learned from them [10]. Whether the discreteness of the bootstrap leads to the generation of more complex graphs as claimed in [10], with more false positive features, or whether it leads to structures with missing edges (false negative features) because of undersampling of weaker relationships in the data, is an open one.

An alternative approach is to use the Bayesian bootstrap [11]. It is a Bayesian resampling procedure that is operationally similar to the ordinary non-parametric bootstrap. In the Bayesian bootstrap, examples are assigned continuously varying weights drawn from a Dirichlet distribution. Not surprisingly, the Bayesian bootstrap procedure has a Bayesian interpretation. Assume that examples are drawn from some unknown multivariate distribution *P*(**X**), and that we have no specific priors on that distribution. The uninformative prior on *P* combined with the multinomial sample likelihood yields, via Bayes Theorem, a Dirichlet posterior distribution on the fraction of the original population that each sampled example represents. The ensemble of Bayesian bootstrap resamples, and the distribution of statistics derived from them, can be viewed as samples from a Bayesian posterior distribution. The continuously varying weights of the Bayesian bootstrap ensure that there is a vanishingly small chance of assigning a zero weight to any example in a resample. Thus, all of the inter-relationships between examples are preserved in the resample, but in reweighted form. Statistics derived from these resamples do not embody the bias introduced by the discreteness of the regular bootstrap.

We now turn to the question of the number of bootstrap resamples needed to form accurate estimates of structure feature probabilities.

### Estimating a bound on the number of bootstrap resamples

The probability of a feature *e* from a bootstrap resample, , will be exactly 0 or 1 if estimated from the single best network. In double averaging, the , although estimated from multiple high-scoring networks, have highly bimodal distributions, each being either close to 0 or close to 1, with few exceptions. Consequently, we here approximate the feature probabilities from each resample as either 0 or 1. The averaged or bagged feature probabilities then have binomial distributions that in the large resample limit approximate a normal distribution with mean *p* and variance(3)

where *p* is the probability of getting a probability value of 1 in each resample, and *B* is the number of resamples. For fixed number of resamples *B*, the largest variance is obtained when *p* = 0.5. The largest feature probability variance can therefore be estimated by(4)

For *B* = 200 (experimental setting used in [4] and suggested in [9]), the largest feature probability variance is 0.25/200 ≈ 0.00125 yielding a standard deviation of approximately 0.035. Three standard deviations around the mean of 0.5 yields a probability range of 0.4 – 0.6. To obtain a more reasonable standard deviation of about 0.01 on link probabilities, and hence a three-sigma range of 0.47-0.53, we need

In practice, we are most concerned about feature probabilities close to the cutoff threshold. For features probabilities *p* greater than 0.5, the number of resamples required to achieve a standard deviation of 0.01 is approximately

Even for a large *p*, for instance 0.9, this works out to be 900 resamples.

In practice, our resample feature probabilities are sometimes not exactly 0 or 1, so we expect the above to be slight overestimates.

## ALARM network simulation study

To understand how different bagging methods, scoring metrics, and their parameters affect learning performance, we conducted an extensive simulation study using the well known ALARM network. This network contains 37 discrete nodes, with two to four values per node, and 46 edges. We drew a master dataset containing 100,000 data points from the ALARM network’s joint probability distribution.

To evaluate the effect of different strategies and parameters on learning performance, we extract a subset from that master dataset and apply the network learning strategy being tested. In all cases, the core learning strategy is the greedy hill-climbing algorithm with random restarts described above. We compute posterior probabilities of network features, such as edges, by determining how often each such feature occurs in the best single network learnt from each bootstrap resample. A slight variation is double averaging, which first computes feature probabilities for each resample by (Bayesian) averaging of multiple high-scoring networks learnt for that resample, and then averages these probabilities across resamples. In either case, the bagging process produces estimates of posterior probabilities  for edge features. Given a threshold 0 ≤ *t* ≤ 1, we will call an edge *e* present if . An edge is a true positive if it is present in the known ALARM network. An edge is labeled a false positive if its posterior probability exceeds *t*, and it is absent in the reference network. An edge is a true negative if its posterior probability is less than *t* and it is absent in the reference network. Edges with posterior probabilities less than *t* that are present in the reference network are false negatives. We do not consider edge direction in our assessments; an edge is defined to be present if the sum of probabilities of occurrence in both directions exceeds the threshold *t*. We report the number of false positives and false negatives as a function of the threshold *t* and use it to assess the quality of models learned. Unless otherwise stated, the learning performance we report are averages obtained by applying the above process to 60 independent subsets of the master dataset.

### Number of bootstrap resamples required

**Figure 3:**
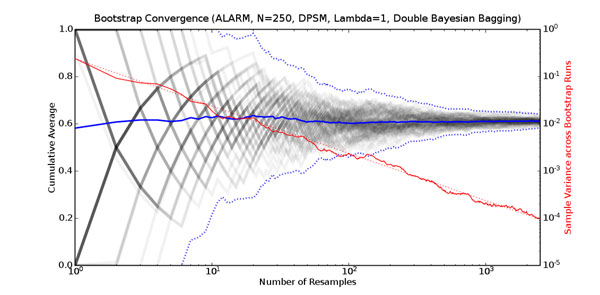
**Bootstrap convergence** This figure shows the convergence of 60 independent bootstrap estimates for the probability of the edge between PULMEMBOLUS and PAP in the ALARM network being present for a single dataset containing 250 data points. The horizontal axis (log scale) is the number of bootstrap resamples. The left-hand vertical axis is the cumulative proportion of top networks that include the edge between PULMEMBOLUS and PAP. Each of the 60 independent bootstrap bagging analyses is plotted as a faint grey line. Overlapping lines are darker in proportion to the number of lines involved. The average across all 60 estimates is plotted as a solid blue line, and the dotted blue lines indicate plus or minus three estimated standard deviations from the average. The right-hand vertical axis (log scale) is the variance of the estimates. The solid red line is the sample variance. The dotted red line is the theoretical estimate according to equation 3.

To estimate the number of bootstrap resamples required, we performed 60 independent Bayesian bootstrap bagging analyses of a single dataset containing 250 data points. Each analysis used identical data, but a different seed for the pseudo-random number generator. To illustrate the convergence of the different bootstrap runs, we choose the edge between PULMEMBOLUS and PAP as a representative example. Figure 3 shows the cumulative probability of this edge being present. Every independent bootstrap run is plotted as a faint grey line. Overlapping lines are darker in proportion to the number of lines involved. The solid blue line is the cumulative average across all bootstrap runs of this posterior probability. The solid red line (on the right-hand side axis) is the variance of the probability across the bootstrap runs. The dotted blue lines indicate three standard deviations (estimated across the bootstrap runs) above and below the sample mean. The dotted red line indicates the expected variance if we assume that the result for each bootstrap resample is either 0 or 1. The variance curve shows that to get a standard deviation of 0.03 to 0.01 for this edge (corresponding to variance of 10^–3^ to 10^–4^), we need between 500 and 2,500 resamples. Most experimental studies in the literature use 200 bootstrap samples, a figure suggested by [9]. However, this experiment (as well as others we have done) indicates we need an order-of-magnitude more resamples for structure learning with limited data. For the remainder of this study, we use 2,500 bootstrap resamples in all experiments.

### Effect of bagging on learning performance

**Figure 4:**
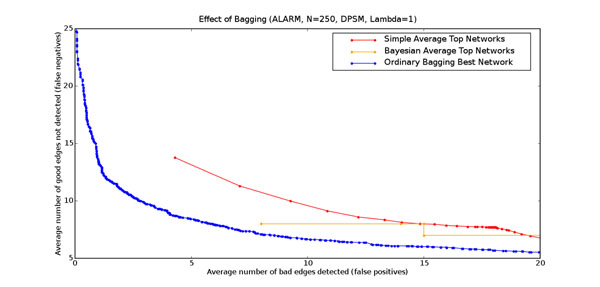
**Effect of bagging on learning performance** This figure shows the effect of bagging on learning performance for the ALARM network. The horizontal axis is the number of false positives and the vertical axis is the number of false negatives. The red curve is computed by simple averaging of the top networks learnt by the search procedure from the original data, while the yellow curve is derived from the same networks weighted by their probability with respect to the data. The blue curve is the simple average of the single best network learnt by the search procedure for each of 2500 bootstrap resamples. The results are averages over 60 independent data sets each containing 250 data points.

Figure 4 shows the impact of bagging on learning performance, for datasets with 250 data points. The scoring function used is DPSM with *λ* = 1. The false positive/false negative tradeoff curve with bagging lies well below the tradeoff curves without bagging. For a fixed number of false positives, bagging yields significantly fewer false negatives. Similarly, for a fixed number of false negatives, bagging yields fewer false positive edges. In the structure learning context, bagging enables us to estimate the posterior probabilities of edge features more accurately, with additional resamples resulting in substantial reduction in variance. This variance reduction translates to more accurate structures learned from the same data. Similar trends are observed for other scoring metrics as well as other bagging approaches.

### Effect of double bagging

**Figure 5:**
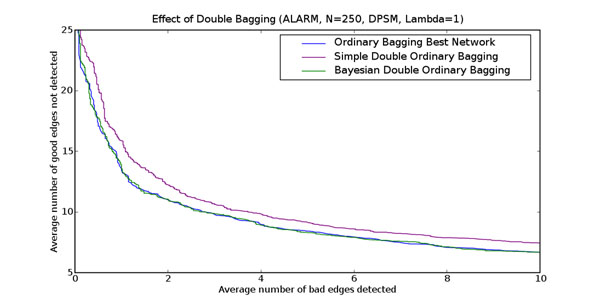
**Effect of double bagging** This figure shows the effect of double bagging on learning performance for the ALARM network. The horizontal axis is the average number of bad edges detected (false positives), and the vertical axis is the average number of good edges not detected (false negatives). The blue curve is the simple average of the single best network learnt by the search procedure for each of the 2500 bootstrap resamples. The magenta curve is the simple average over 2500 bootstrap resamples of the simple average of the best ten networks learnt by the search procedure for each resample. The green curve is the simple average over 2500 bootstrap resamples of the Bayesian average of the best networks learnt by the search procedure for each resample. Each curve is the average of 60 independent data sets each containing 250 data points.

The learning procedure, with its multiple restarts, often produces several top networks with very similar high scores. Since the greatest computational effort, by far, is the learning procedure, it might be possible to improve learning performance by deriving a composite probability for each feature from several of these top scoring networks from each resample. We compared bagging using only the best network, to bagging of feature probabilities estimated by a simple average and a Bayesian average of multiple, distinct high-scoring networks from each bootstrap resample. This averaging over the top networks occurs prior to bagging, as shown on the right hand side of Figure 2. Figure 5 shows the results of double averaging versus picking the single best on the ALARM data set with scoring function DPSM (*λ* = 1).

### Effect of bias-correction strategies on model accuracy

**Figure 6:**
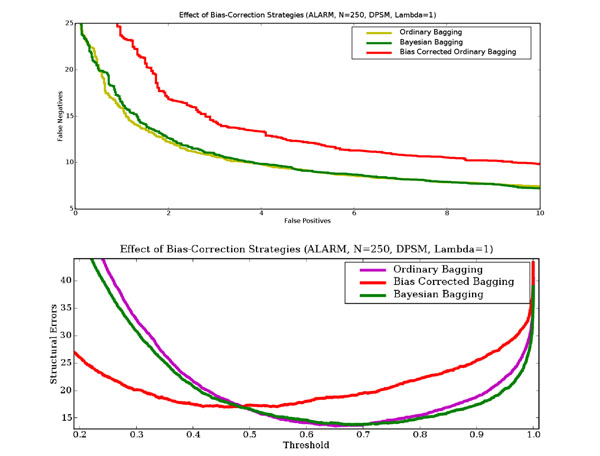
**Effect of bias-correction strategies** This figure shows the effect of bias correction strategies on learning performance for the ALARM network. In the upper panel, the horizontal axis is the number of false positives and the vertical axis is the number of false negatives. In the lower panel, the horizontal axis is the probability threshold above which an edge is deemed to be present, and the vertical axis is the total number of errors (false positives plus false negatives). The results are averages over 60 independent data sets each containing 250 data points. The DPSM scoring metric with *λ* = 1 was used.

To determine the impact of bias introduced by discrete bootstrap resampling, and the effect of changes to the scoring function to account for it, we compared ordinary bagging, ordinary bagging with a bias corrected scoring function [10], and Bayesian bagging on the same ALARM data set with 250 points. The upper panel in figure 6 shows the false positive/false negative tradeoff curves for the three bagging approaches. It clearly shows that the bias corrected scoring metric performs worse than ordinary and Bayesian bagging. The lower panel in figure 6, which shows the number of structural errors (false positives and false negatives) as a function of threshold choice, reinforces this result. Similar trends are observed for different dataset sizes and different choices of scoring functions.

Below we offer a possible explanation for this result. Ordinary bagging and Bayesian bagging appear to have very similar performance, with Bayesian bagging outperforming ordinary bagging in terms of the number of structural errors for thresholds above 0.72. Since this threshold range is likely to be the range of greatest interest, especially in the context of limited data, we believe that Bayesian bagging is a better choice for structure learning of Bayesian networks.

### Effect of local scoring function

**Figure 7:**
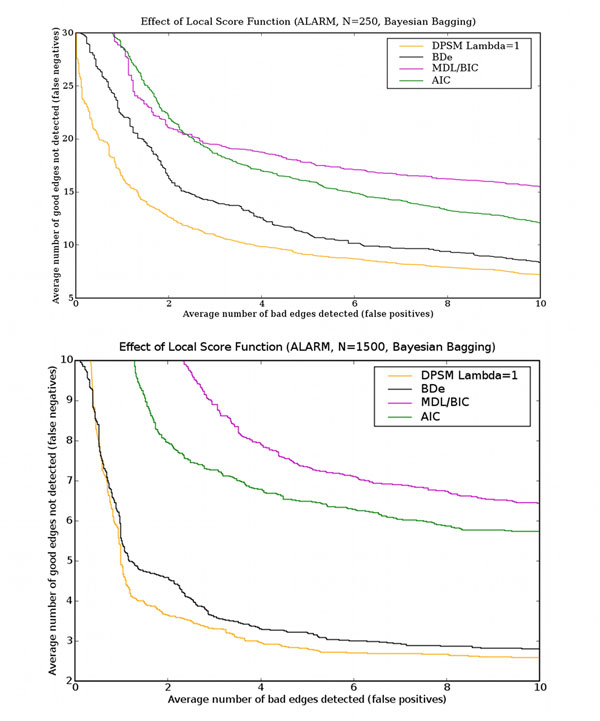
**Effect of local scoring function** This figure shows the effect of the scoring metric on learning performance for the ALARM network. The horizontal axis is the number of false positives and the vertical axis is the number of false negatives. The results are averages over 60 independent data sets each containing either 250 (top panel) or 1500 (bottom panel) data points. Bayesian bagging was used.

Figure 7 shows the impact of the scoring metric on learning performance, using Bayesian Bagging on datasets with 250 data points. Similar trends are observed for datasets of other sizes. Figure 7 makes it clear that MDL/BIC performs very poorly, and also confirms Hartemink’s result [16] that BDe performs better than MDL/BIC. Surprisingly, DPSM with *λ* = 1 performs the best. As the next section shows, DPSM with values of *λ* less than 1 perform even better. This surprising experimental result reveals an important requirement for scoring functions to be effective with bagging, particularly in contexts with limited data.

### Effect of *λ* for DPSM scoring function

**Figure 8:**
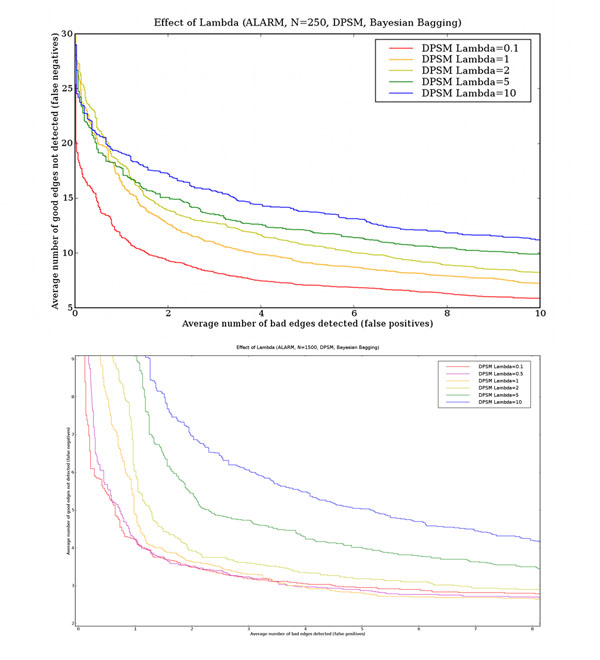
**Effect of *λ* for DPSM scoring function** This figure shows the effect of *λ* on learning performance for the ALARM network. The horizontal axis is the number of false positives and the vertical axis is the number of false negatives. The results are averages over 60 independent data sets each containing either 250 (top panel) or 1500 (bottom panel) data points. Bayesian bagging and the DPSM scoring metric were used.

Figure 8 shows the impact of *λ* on the performance of the DPSM scoring metric on ALARM. Learning performance, measured in terms of the false positive/false negative tradeoff curve continues to improve as *λ* is reduced until approximately 0.1 to 0.2, below which learning performance rapidly deteriorates. In subsequent experiments, we therefore use the DPSM metric with *λ* = 0.1.

As *λ* values are lowered, the edge density of the Bayesian averaged graphs produced from each bootstrap resample increases. Our results suggest that it is a good strategy to learn overfitted models from each bootstrap resample, and to let bagging average out feature probability estimates of false positive edges to zero. The benefit of learning overfitted models is the reduction in the number of false negative edges. This explains why applying a bias correction factor to the scoring metric (see above) reduces learning performance. While bias correction ensures that false positive features are minimized, it increases the number of false negative features because of its conservatism in adding edges. Thus the total number of structural errors is higher with bias corrected scoring functions than with DPSM with *λ* = 0.1.

Our experimental results suggest that when data is limited, promiscuous scoring functions (such as DPSM with *λ* < 1) are very effective when combined with Bayesian bagging over a sufficiently large number of bootstrap resamples. Both the false positive and false negative rates decrease as a result of investment of additional computational resources, and not additional data.

### Effect of training set size

**Figure 9:**
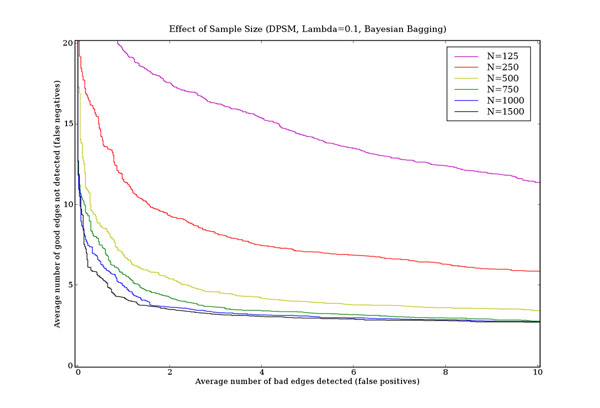
**Effect of training set size** This figure shows the effect of training set size on learning performance for the ALARM network. The horizontal axis is the number of false positives and the vertical axis is the number of false negatives. The results are averages over 60 independent data sets each containing 250 data points. The DPSM scoring metric with *λ* = 0.1 was used.

Figure 9 shows the impact of sample size on learning performance, using Bayesian bagging and the DPSM (*λ* = 0.1) scoring metric. As expected, increasing sample size improves learning performance. This improvement is non-linear, with smaller sample sizes showing much greater relative improvement than larger sample sizes. For this data set, increases in dataset size up to 500 data points show the greatest improvements, although it is possible to learn many edges from datasets with as few as 125 data points. For the ALARM network, sample sizes under 500 qualify as limited data scenarios, where Bayesian bagging with promiscuous scoring functions offers the greatest benefit in terms of learning performance.

### Comparison to other methods

**Figure 10:**
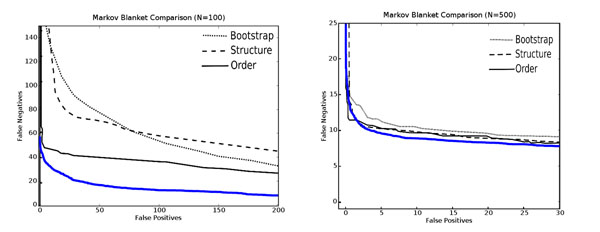
**Comparison to other methods** This figure compares the learning performance of our method (in blue) with the ones presented by Friedman and Koller in the top two panels on the left side of Figure 11 in [13] (in black).

Figure 10 compares Bayesian Bagging using the DPSM(*k* = 8, *λ* = 0.1) metric to the methods reported by Friedman and Koller [13]. Our method is significantly better for small datasets (N=100). There are three key differences in our model-averaging approaches. Friedman and Koller use ordinary bagging with 200 bootstrap resamples, the BDe scoring function, and an order-based MCMC search algorithm. We use Bayesian bagging with 2500 bootstrap resamples and a greedy search algorithm with randomized restarts based on the sparse-candidate algorithm (*k* = 8), and the DPSM scoring function with *λ* = 0.1. Figure 10 clearly indicates the reduction in the number of false negative features in our approach. As we conjectured before, we attribute this reduction to the construction of overfitted models in each bootstrap resample run and the use of bagging over 2500 resamples to eliminate false positive features. When sample sizes get larger, the extra investment of computation does not pay off as much, as indicated on the right hand panel of Figure 10.

**Figure 11:**
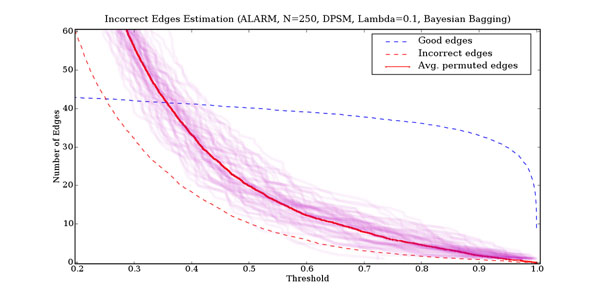
**Threshold selection using permutation testing** Estimating the number of incorrect edges from the number of edges found in permuted data sets for the ALARM network. The horizontal axis is the frequency threshold above which an edge is said to be present. The vertical axis is the number of edges. The dashed blue line is the number of correct edges. The dashed red line is the number of incorrect edges. The faint purple lines are the number of edges found for each of 60 independent permuted data sets. Overlapping lines are darker in proportion to the number of lines involved. The solid red line is the average number of edges found in the permuted data sets.

## Threshold selection using permutation testing

When we attempt to learn a network structure using the above bagging procedure, the edge frequencies obtained will range between 0 and 1. We need a global threshold on edge frequencies to determine whether or not an edge is to be included in the final model. The optimal threshold to use is not constant, but depends on the specific dataset being analyzed. Consequently, in practice, when learning a network model from data, both the true model and the optimal threshold to use are unknown. We need a method to estimate the threshold that minimizes the number of structural errors made (false positive and false negatives).

Given randomly permuted (null) data, our bagging process will compute edge frequencies. Our hypothesis is that the edge frequencies of incorrect edges are similar to the edge frequencies obtained from randomly permuted data. Consequently, the number of edges found in a randomly permuted data set that are above a particular threshold will be indicative of the number of incorrect edges above that threshold in the network learnt from the unpermuted data. Our permutation test determines the likely number of incorrect edges above a particular threshold by averaging the number of edges above that threshold across 60 random permutations of the data. If the data is in fact random, this is clearly a reasonable estimate. For non-random data, it appears that the number of edges obtained from the permuted data overestimates the number of incorrect edges (see figure 11). Consequently, we believe that the number of edges in permuted data is a conservative but reasonable estimate of the number of incorrect edges.

**Figure 12:**
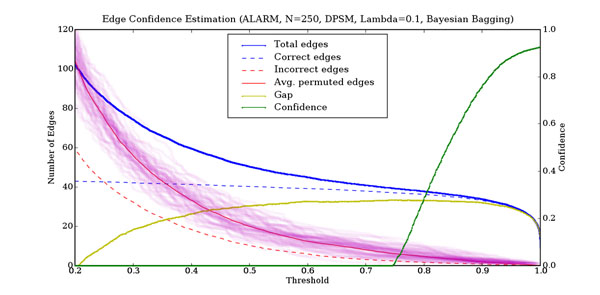
**Edge confidence estimation for the ALARM network** The horizontal axis is the frequency threshold above which an edge is said to be present. The left-hand vertical axis is the number of edges. The solid blue line is the total number of edges. The faint purple lines are the number of edges found for each of 60 independent permuted data sets. The solid red line is the average number of edges found in the permuted data sets. The solid yellow line is the difference between the total number of edges and the average number of edges in the permuted datasets. The right-hand vertical axis is the confidence that an edge is real. The solid green line is the confidence associated with an edge found at the threshold concerned.

To select a threshold, we compute the difference between the total number of edges found and the estimated number of incorrect edges found by the permutation test. A simple threshold, however, does not discriminate between edges that are way above the threshold (and very unlikely to be incorrect) from those just above it (and much more likely to be incorrect). Consequently, for edge frequencies (*f*) above the threshold, we compute an edge likelihood (*L_f_*) from the slope of total number of edges (Δ*t_f_*) and the slope of estimated number of incorrect edges (Δ*p_f_*) at that edge frequency

with the added constraint that it monotonically decreases as the threshold decreases. Since neither the total number of edges nor the number of permuted edges are smooth functions, we approximate a smooth result by averaging finite differences over a range of widths. Figure 12 plots our estimated edge confidence as a function of the threshold for an instance of the ALARM network.

## INSURANCE network simulation study

To determine the generality of the above results we performed a similar study using the INSURANCE network. As was the case for the ALARM network, learning performance using DPSM improves as *λ* decreases, but in this case the best performance was obtained using BDe.

## Application to biological dataset

**Figure 13:**
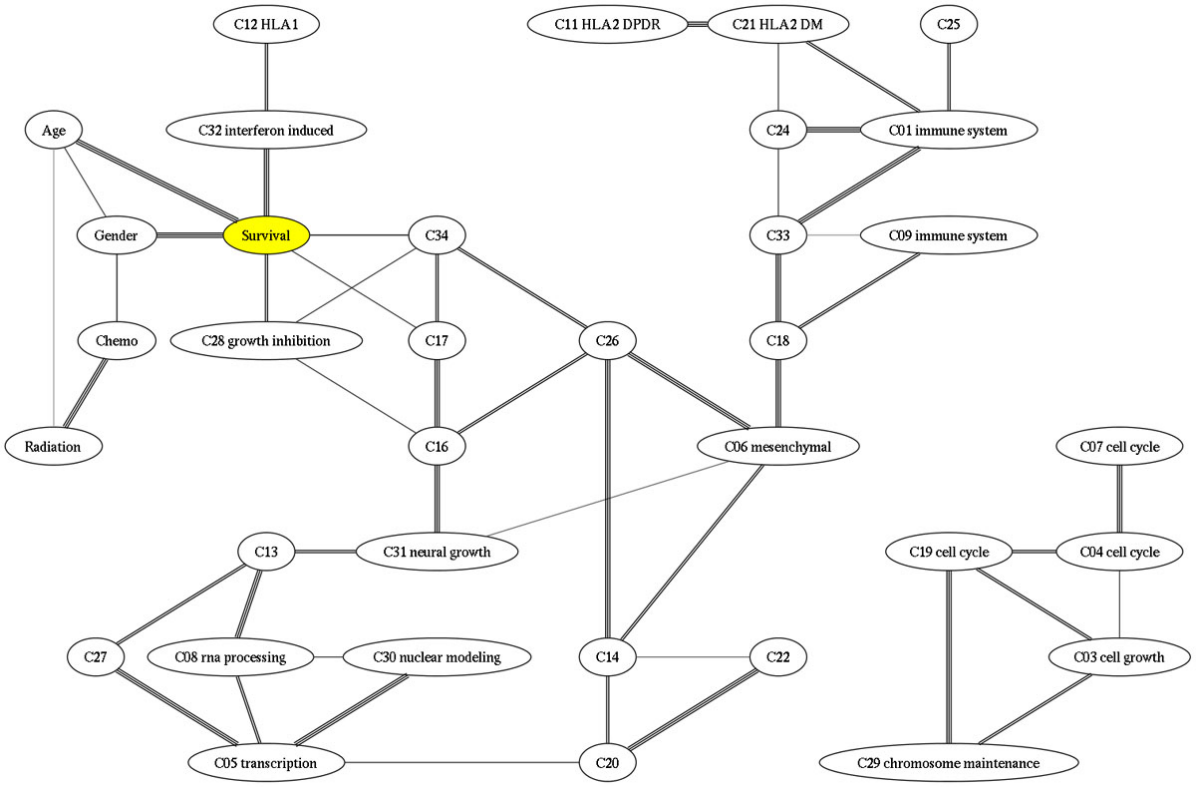
**Consensus Bayesian Network of a Glioblastoma Dataset** The consensus Bayesian network obtained by applying our method to the Glioblastoma dataset. The color of each edge indicates the frequency that that edge occurred in the bagged resamples.

Figure 13 shows the consensus Bayesian network obtained by applying our method to the Glioblastoma dataset [22].

We use bagged gene shaving [23] to extract consensus gene clusters. Clusters containing genes with an obvious common biological function were also given a descriptive name. To generate a Bayesian network, we trichotomized the signed mean gene for each cluster into low, medium, and high values. We included additional nodes for clinical covariates of interest, including age, gender, whether chemotherapy or raditional was given and survival. We dichotomized survival into short and long survivors at 12 months, but marked as not available all censored survival times shorter than 12 months.

According to our analysis, survival is linked most closely with two clinical covariates, age and gender, and two gene clusters, a cluster of interferon induced genes and a cluster of growth inhibition genes.

## Conclusions and future work

We explored the space of model-averaging strategies in contexts with limited data with the goal of robustly learning large Bayesian networks. Our results follows.

1. *Is bias-correction a good idea?* In contexts with limited data, Bayesian bagging with scoring functions that permit the learning of overfitted models is a better strategy than using model-averaging with conservative scoring functions. In our experiments, bias-corrected scoring functions with Bayesian bagging have higher number of structural errors, both in terms of false positives (incorrect features) and false negatives (missing features).

2. *Which is better: Bayesian bagging or ordinary bagging?* Bayesian bagging yields models with more features than ordinary bagging. At high feature selection threshold values, Bayesian bagging yields better models, especially when sample sizes are small. As sample sizes increase, the two approaches are nearly indistinguishable in performance as measured by the number of false positives and false negatives. In limited data contexts, Bayesian bagging is superior to ordinary bagging.

3. *How many bootstrap resamples are needed?*: Our experiments show that for features whose true probability is close to the threshold for inclusion in the final model, up to 2500 bootstrap samples are needed for robust estimation. Previous studies [7,10] have used one to two hundred bootstrap resamples. Noise features (false positives) are averaged out by bagging over such a large ensemble. This improvement comes not at the expense of false negatives, which are also reduced by the protocol.

4. *Which class of scoring functions yield the most accurate models?*: For the ALARM network, the DPSM scoring metric with small values of *λ* performed best, whereas for the INSURANCE network the BDe metric performed best. What both of these metrics have in common is that they very readily include additional edges into the network. A promiscuous scoring metric that produces overly complex models works well in the context of bagging because it is more likely to include true edges with weak support in each resample, hence significantly reducing false negatives, while the bagging method effectively eliminates the false positives introduced by the scoring metric.

5. *How is a feature selection threshold to be determined?*: We developed a permutation based method to compute significance thresholds for feature selection in bagged graphs. In the absence of a true model, the threshold gives us a bound on the number of false positive features in the final graph.

6. *How many data samples are needed?*: Dataset size is a key determiner of performance of the learning methods. Our model averaging strategy with bayesian bagging, and the DPSM scoring function (*λ* = 0.1) with feature selection using a threshold estimated by permutation testing, outperforms existing methods and yields excellent models even with samples sizes as low as 125 for the ALARM network. This result suggests that our model averaging approach is suitable for biological applications such as learning regulatory genetic networks, characterized by several tens of nodes and training data sets of the order of a few hundred samples.

One of the open questions is a theoretical characterization of the relationship between model complexity and sample size for Bayesian networks. In this paper, we characterized this relationship empirically for two well-known networks of moderate complexity. Our model averaging strategy learns more accurate models than other approaches when the amount of data is limited relative to the complexity of the model being learned. In future work we plan to explore more networks in biological applications, and refine our protocol for learning high confidence models with limited data.

## List of abbreviations used

AIC: Aikake Information Criterion; BDe: Bayesian Dirichlet metric; BIC: Bayesian Information Criterion; DPSM: Dirichlet Prior Scoring Metric; GBM: Glioblastoma Multiforme; MCMC: Markov Chain Monte Carlo; MDL: Minimum Description Length; TCGA: The Cancer Genome Atlas; UPSM: Uniform Prior Scoring Metric.

## Competing interests

The authors declare that they have no competing interests.

## Authors' contributions

BB and DS conceived of the study, and participated in its design and coordination and helped to draft the manuscript. KA consulted on statistical considerations. All authors read and approved the final manuscript.
